# Supplementary material for: Rewilding Apex Predators Has Effects on Lower Trophic Levels: Cheetahs and Ungulates in a Woodland Savanna
Source: Animals (Basel). 2022 Dec 14;12(24):3532. doi: 10.3390/ani12243532 (PMC9774585; doi:10.3390/ani12243532)

Supplementary Information:

Dallas B. Ruble, Stijn Verschueren, Bogdan Cristescu* and Laurie L. Marker. Rewilding Apex Predators Has Effects on Lower Trophic Levels: Cheetahs and Ungulates in a Woodland Savanna. *Animals*.

* bogdan@cheetah.org

Supplementary Table S1: Frequency of cheetahs released in Bellebenno A per year with waterhole survey occurrence.

Year	Releases	Waterhole survey occurrence
2004	3	No
2005	1	No
2006	0	No
2007	3	No
2008	5	Yes
2009	0	Yes
2010	4	Yes
2011	5	Yes
2012	14	Yes
2013	1	Yes
2014	5	Yes

Supplementary Table S2: The average (x̄) and standard deviation (*SD*) of individual ungulate, herd records, and duration of stay at waterholes during treatment 1 (2008-2014) and treatment 0 (2015-2019).

		Individual ungulate		
Treatment	Estimates	Small	Medium	Large
Cheetah presence	x̄	0.49	0.02	2.00
	SD	1.16	0.15	4.28
Cheetah absence	x̄	0.60	1.13	0.80
	SD	1.25	3.71	2.82

Herd records

Treatment	Estimates	Small	Medium	Large
Cheetah presence	x̄	0.44	0.02	0.79
	SD	1.02	0.15	1.67
Cheetah absence	x̄	0.48	0.30	0.35
	SD	1.00	0.79	0.97

Duration of stay

Treatment	Estimates	Small	Medium	Large
Cheetah presence	x̄	17.48	5.50	17.69
	SD	21.67	6.36	17.48
Cheetah absence	x̄	13.23	7.31	9.24
	SD	13.62	6.58	10.49

Supplementary Table S3: BIC random effect and fixed effect model estimates to determine best fit model for individual visitation rate by body size.

	Random effect	Fixed effect
	BIC	BIC
Negative Binomial	803.342	797.274
Zero-inflated Poisson	870.880	870.024
Poisson	1591.418	1590.925
Goodness of fit analysis	Pr(>Chisq)	Pr(>Chisq)
Negative Binomial	1.000	

Supplementary Table S4: BIC random effect and fixed effect model estimates to determine best fit model for herd record visitation rate.

	Random effect	Fixed effect
	BIC	BIC
Negative Binomial	674.988	673.115
Zero-inflated Poisson	679.184	668.919
Poisson	830.217	824.149
Goodness of fit analysis	Pr(>Chisq)	Pr(>Chisq)
Negative Binomial	1.000	

Supplementary Figure S1: Duration of waterhole visits for small, medium, and large ungulates in response to cheetah presence (treatment 1) or absence (treatment 0).

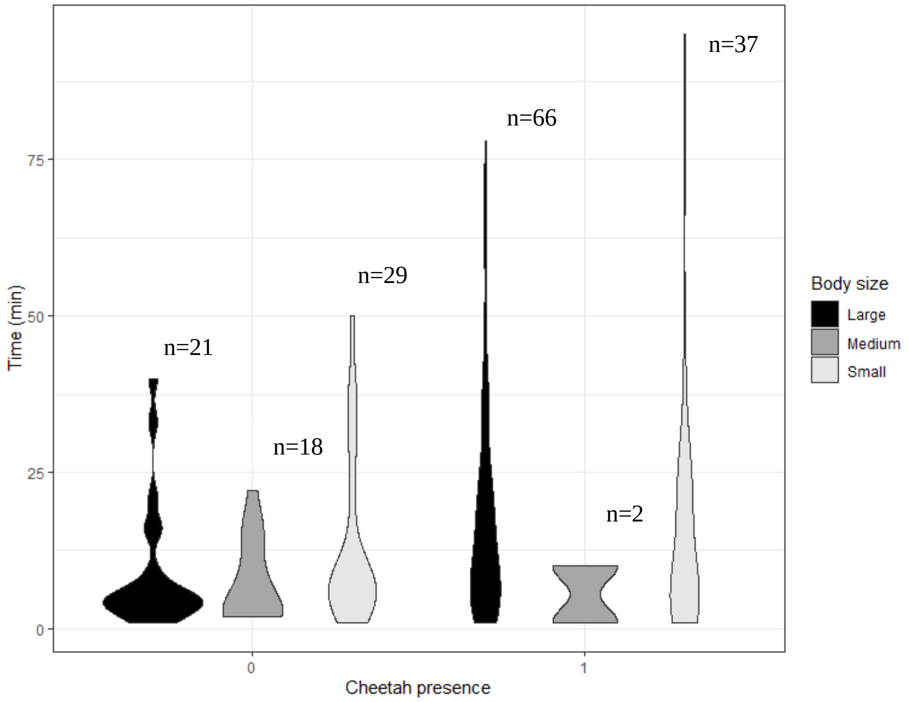

Supplementary Figure S2: Activity overlap among ungulates during cheetah presence (treatment 1) and absence (treatment 0). Small, medium, and large ungulates are pooled. The x-axis ticks indicate ungulate observation records. The shaded area below the two curves illustrates the overlap in ungulate activity patterns between the two treatments. Dhat1 is the coefficient of overlap.

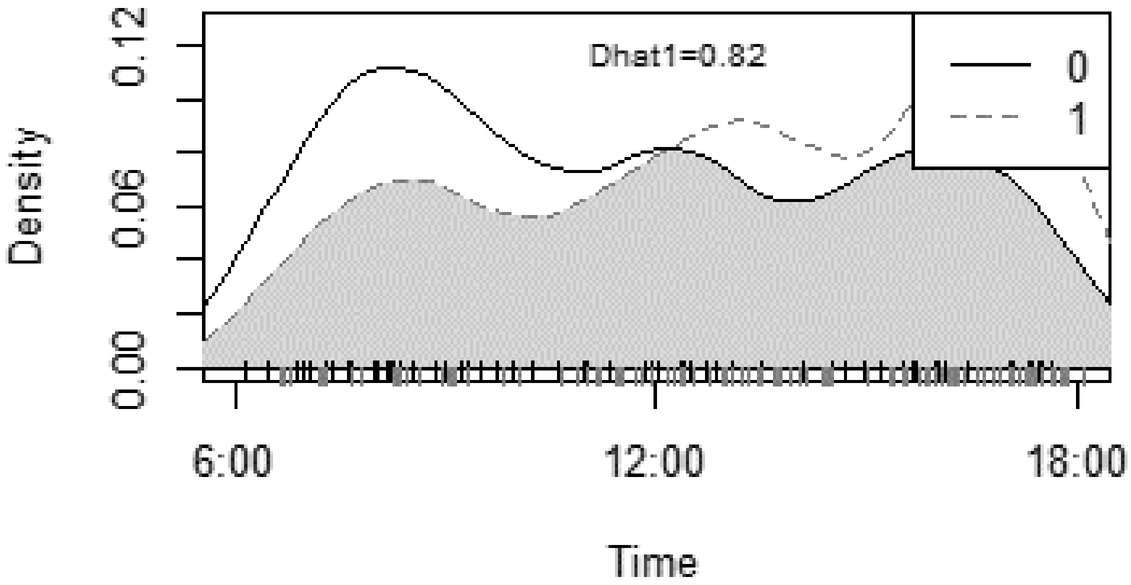

Supplement: Supplementary file 1 [file animals-12-03532-s001.zip › animals-2071544-supplementary.pdf]
